# “But His Yelp Reviews Are Awful!”: Analysis of General Surgeons’ Yelp Reviews

**DOI:** 10.2196/11646

**Published:** 2019-04-30

**Authors:** Cynthia Liu, Meka Uffenheimer, Yosef Nasseri, Jason Cohen, Joshua Ellenhorn

**Affiliations:** 1 The Surgery Group of Los Angeles Research Foundation Los Angeles, CA United States

**Keywords:** patient satisfaction, general surgery, Los Angeles, Web-based ratings, digital health, Yelp

## Abstract

**Background:**

Patients use Web-based platforms to review general surgeons. However, little is known about the free-form text and structured content of the reviews or how they relate to the physicians’ characteristics or their practices.

**Objective:**

This observational study aimed to analyze the Web-based reviews of general surgeons on the west side of Los Angeles.

**Methods:**

Demographics, practice characteristics, and Web-based presence were recorded. We evaluated frequency and types of Yelp reviews and assigned negative remarks to 5 categories. Tabulated results were evaluated using independent *t* test, one-way analysis of variance, and Pearson correlation analysis to determine associations between the number of total and negative reviews with respect to practice structure and physician characteristics.

**Results:**

Of the 146 general surgeons, 51 (35%) had at least 1 review and 29 (20%) had at least 1 negative review. There were 806 total reviews, 679 (84.2%) positive reviews and 127 (15.8%) negative reviews. The negative reviews contained a total of 376 negative remarks, categorized into physician demeanor (124/376, 32.9%), clinical outcomes (81/376, 22%), office or staff (83/376, 22%), scheduling (44/376, 12%), and billing (44/376, 12%). Surgeons with a professional website had significantly more reviews than those without (*P*=.003). Surgeons in private practice had significantly more reviews (*P*=.002) and more negative reviews (*P*=.03) than surgeons who were institution employed. A strong and direct correlation was found between a surgeon’s number of reviews and number of negative reviews (*P*<.001).

**Conclusions:**

As the most common category of complaints was about physician demeanor, surgeons may optimize their Web-based reputation by improving their bedside manner. A surgeon’s Web presence, private practice, and the total number of reviews are significantly associated with both positive and negative reviews.

## Introduction

One of the most ubiquitous business review websites, Yelp was established because the founder was unable to find recommendations for local physicians on the Web [1]. As customer feedback has become increasingly accessible, Web-based rating sites now have an influential impact on the impression and decisions of patients, with as many as 68% of patients turning to these resources to research or review physicians [2]. Physicians are beginning to realize that reactions and ratings detailed on these websites may impact which and how many patients visit them, as well as their overall reputation [3-5].

There are at least 33 websites where patients can describe their experience at hospitals, clinics, or clinical practices [6]. These websites range from general consumer rating websites to websites geared specifically toward the medical field. The structure of the all-purpose websites tends to afford more freedom to the commenters, whereas medical-based websites generally have a more structured format. In addition to premade surveys, several medical-based rating websites also allow reviewers to make unique remarks [6]. Yelp is one of the most used Web-based resources to review physicians [7-12].

An analysis of the content of Web-based reviews of general surgeons, including free-form content, has not been systematically described. We investigated the Yelp reviews of general surgeons in a defined region to categorize the content of the negative reviews and determine whether the number of reviews and the number of negative reviews correlated with the characteristics of the physicians. Los Angeles was chosen as the site of this study because it is home to a large variety of practices and institutions.

## Methods

We identified general surgeons practicing on the west side of Greater Los Angeles using The Medical Board of California Web-based database [13] and InfoUSA (Papillion, Nebraska), a marketing company that provides contact databases and mailing lists. The physicians practicing in the 31 zip codes on the west side of Los Angeles were examined. The active practice status of the physician was determined using The Medical Board of California’s License verification Web-based tool [13]. Those surgeons who were still in training, retired, and those without an active medical license were eliminated. Physician gender, years since graduating from medical school, and medical school attended were identified. A Google search was performed to determine if the physician had a medical practice website.

A physician was considered to have a Yelp page if they could be found and reviewed on a page designated for an institution, clinical practice, or the physician. The following was documented: presence of a Yelp page, number of reviews, number of positive reviews, and number of negative reviews. Yelp users rate an institution or physician on a 5-star system, with 5 stars defined as “Woohoo! As good as it gets!” and 1 star defined as “Eek! Methinks not.” Yelp defines 4 stars as “Yay! I’m a fan.” As 4 stars was less than a perfect star rating, a negative review was defined as a review that contained at least 1 negative remark and had a rating of 4 stars or fewer. Yelp defines 3 stars as A-OK, and because it is not a 4- or 5-star review, a level of mediocrity is implied [14]. A positive review was defined as any 5-star or 4-star review that contained no negative remarks.

Negative reviews were further characterized. Each commenter’s username, date of review, and star rating was noted. Free-form reviews were manually tabulated, categorized, and resolved by 2 independent reviewers and were empirically divided into 5 categories modified from previously defined categories [15]: Scheduling (doctor availability and punctuality), billing, office and staff (staff friendliness or professionalism, staff presence, and office décor or location), clinical outcome (correct diagnosis or treatment, technical skill, treatment of unforeseen complications, if additional treatment was needed, and follow-up care), and physician demeanor (education, empathy, bedside manner, professionalism, preparedness or organization, time with doctor, communication skills, shared decision making, and general impression). Remarks within negative reviews were noted to be negative, neutral, or positive.

Once the raw data were collected, summary statistics were calculated using univariable analyses. An independent *t* test, which determines whether there is a statistically significant difference between the means in 2 unrelated groups, was run to determine whether or not certain factors (possession of professional website, private practice or institution employed, gender, and medical school outside of the United States) impacted the number of total reviews or negative reviews of a surgeon. One-way analysis of variance analysis, which determines whether there is a statistically significant difference between the means in more than 2 unrelated groups, was used to determine if zip code or the number of years since graduating from medical school impacted a surgeon’s number of total reviews or negative reviews. A Pearson correlation, which measures the linear correlation between 2 variables, was performed to determine if there was any relationship between a give surgeon’s total number of reviews and quantity of negative reviews. Statistical Package for the Social Sciences statistical software was used.

## Results

We identified 146 practicing general surgeons on the west side of Greater Los Angeles. A total of 33 (22.6%, 33/146) surgeons were female and 113 (77.4%, 113/146) were male. Moreover, 55 (37.7%, 55/146) surgeons were in private practice and 91 (69.2%, 91/146) were institution employed. In addition, 19 (13.0%, 19/146) surgeons went to medical school outside of the United States. Furthermore, 42 (28.8%, 42/146) physicians had a professional website (Table 1).

**Figure 1:**
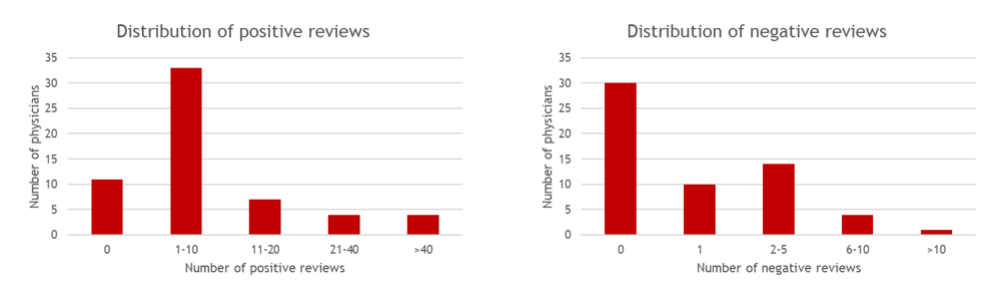
Distribution of reviews. The chart on the left shows the breakdown of positive reviews among the 59 physicians who had a Yelp page, whereas the chart on the right shows the breakdown of negative reviews. The number of negative and positive reviews is shown along with the number of physicians who had that number of negative and positive reviews, respectively.

A total of 59 (40.4%, 59/146) surgeons had a Yelp page. Moreover, 51 physicians (34.9% of all surgeons and 86% of those with a Yelp) had at least 1 review. Of the 59 physicians who had a Yelp page, 29 (49%, 29/59) had at least 1 negative review and 48 (81%, 48/59) had at least 1 positive review (Figure 1).

There was a total of 806 documented reviews. Of these reviews, 679 (84.2%) were positive and 126 (15.6%) were negative. Within the 126 negative reviews, there were 376 negative remarks: 124 (32.9%) concerning physician demeanor, 81 (21.5%) on clinical outcomes, 83 (22.0%) regarding the office or staff, 44 (11.7%) about scheduling, and 44 (11.7%) in relation to billing (Table 1).

**Table 1:** Demographics (N=146).

Characteristics	Statistics, n (%)
Male	113 (77.4)
Private practice	55 (38)
Institutional employed	91 (69.2)
Physicians with website	42 (28.7)
Foreign medical school graduate	19 (13.0)
Physicians with a Yelp page	59 (40.4)
Physicians reviewed on Yelp	51 (34.9)
Physicians with negative reviews	29 (19.8)

**Table 2:** Statistical analysis via 2-way Pearson correlation analysis.

Reviews	Number of reviews, mean (SD)	*P* value
Total reviews (n=806)	5.6 (18.08)	.001
Negative reviews (n=126)	0.86 (4.33)	.001

The existence of a professional website as well as the type of employment had a significant impact on the total number of reviews (Tables 2-4). Those with a professional website had significantly more overall reviews compared with those without (mean 16.36, SD 31.0 vs mean 1.14, SD 3.23; *P*=.003). Two-thirds of physicians in private practice had reviews, compared with one-third of physicians employed by an institution (mean 18.0, SD 31.4 vs mean 0.96, SD 3.18; *P*=.002). Being in private practice was also significantly associated with the number of negative reviews (mean 3.11, SD 8.13 vs mean 0.09, SD 0.35; *P*=.03). As such, private practice was significantly associated with both more overall reviews and more negative reviews. Furthermore, there was a trend toward a correlation between a greater number of reviews and a greater number of years since graduation from medical school (*P*=.05).

A 2-tailed Pearson correlation was performed to evaluate the association between the total number of reviews and the number of negative reviews. The correlation between the total number of reviews and negative reviews was strong (*r*=.862; *P*<.001).

The total number of reviews was not impacted by a surgeon’s gender (male: mean 4.2, SD 11.6 vs female: mean 9.9, SD 31.3; *P*=.32), whether they attended medical school outside the United States (non-United States: mean 1.6, SD 3.20 vs United States: mean 6.1, SD 19.3; *P*=.32), or the practice zip code (*P*=.20).

The likelihood of having negative reviews did not significantly differ between surgeons with a professional website versus surgeons without a professional website (mean 2.3, SD 7.80 vs mean 0.28, SD 0.98; *P*=.10). Similarly, the number of negative reviews was not significantly affected by a surgeon’s gender (male: mean 0.48, SD 1.22 vs female: mean 2.18, SD 8.79; *P*=.28), whether or not they attended medical school outside the United States (non-United States: mean 0.6, SD 1.54 vs United States: mean 0.91, SD 4.6; *P*=.76), the zip code they practiced in (*P*=.77), or the number of years since the surgeon graduated from medical school (*P*=.85).

**Table 3:** Statistical analysis via independent *t* test.

Variable	Number of physicians with any reviews	Reviews			Negative reviews		
		Total, N	Mean (SD)	*P* value	Total, N	Mean (SD)	*P* value
Private practice physicians (n=55)	34	720	18 (31.4)	.002	118	3.11 (8.13)	.03
Institution employed physicians (n=91)	17	86	0.96 (3.18)	.002	8	0.09 (0.35)	.03
Medical school out of United States (n=19)	5	31	1.6 (3.20)	.32	11	0.6 (1.54)	.76
Medical school in United States (n=127)	46	775	6.1 (19.3)	.32	115	0.91 (4.60)	.76
Male (n=113)	38	480	4.2 (11.6)	.32	54	0.48 (1.22)	.28
Female (n=33)	13	326	9.9 (31.3)	.32	72	2.18 (8.79)	.28
Possess professional website (n=42)	28	687	16 (31.0)	.003^a^	97	2.3 (7.80)	.10
Do not possess professional website (n=104)	23	119	1.1 (3.23)	.003	29	0.28 (0.98)	.10

**Table 4:** Statistical analysis via 1-way analysis of variance analysis.

Variable	*P* value	
	Comparing total number of reviews	Comparing number of negative reviews
Number of years since graduating from medical school	.05	.85
Zip code	.20	.7

## Discussion

Our finding that the majority of Web-based reviews were positive supports prior findings [10,16]. Unfortunately, negative reviews do exist and can have a damaging effect on a physician’s reputation and practice [3,5,9,17,18]. The most common category of Yelp complaints was physician demeanor. As such, surgeons may optimize their Web-based reputation by improving their bedside manner. A surgeon’s type of practice (ie, private practice or institution employed) and the total number of reviews were significantly associated with more negative reviews. A surgeon’s type of practice and the possession of a personal website were significantly associated with more total Yelp reviews.

Of the negative reviews, the most common category was criticism of physician demeanor. Patient perception of physician demeanor is a factor about which physicians may have some control. Thus, it might be possible to improve one’s reviews by enhancing patient-physician interactions. This includes being better prepared for each consultation, spending sufficient time with patients, clearly communicating the plan of care and disease processes affecting each patient, and displaying empathy. Over one-fifth of the complaints centered around clinical outcomes and another one-fifth concentrated on the office or staff. Although physicians are not always in control of clinical outcomes, keeping one’s skills up to date and practicing evidence-based medicine may improve clinical outcomes. To reduce the number of negative Web-based reviews, it is probably important to treat unforeseen complications quickly and empathically. In addition, hiring courteous office staff and optimizing the aesthetic appearance of one’s practice environment may enhance the quality of Web-based reviews.

Our study found that Web presence significantly impacted a physician’s total number of reviews. Presence of a website, personal or private practice, was shown to have a significant effect on the total number of reviews a physician received. The mean number of reviews for those with a professional website was significantly greater than the number of reviews for surgeons without a website, indicating that an increased Web presence leads to more Web-based reviews. General surgeons in private practice had significantly more reviews overall (*P*=.002) and significantly more negative reviews (*P*=.03) than those who were institution employed. In addition, the total number of reviews was strongly correlated with the number of negative reviews. This suggests that although a Web-based presence may be important in enhancing a surgeon’s reputation, it may also be detrimental, depending on the content of the individual reviews.

There are several limitations to this study. Restricting physicians examined to a single geographic area is descriptive for the region but is also limiting in size and scope. Due to the presence of large health institutions in the west side of Los Angeles, the number of surgeons with reviews may be artificially low because institution-based physicians may have a much more limited Web presence and thus fewer Web-based reviews. Yelp was the only Web-based rating website examined, excluding any reviews and Web-based presence surgeons may have had on other rating websites [7]. There is inherent response bias as patients make the active decision whether to write a review or not. This study only aimed to examine patient perception of physicians as well as any perceived problems they encountered in their experiences, and as such, we cannot comment on how this reflects on the quality of the physicians themselves [17,19].

Future studies are needed to determine if the trends and correlations found are applicable to surgeons practicing in other and larger geographic regions. Other specialties need to be examined to see if there is a difference in the number and type of reviews and Web presence among other specialties. Most importantly, the impact of Yelp reviews on a surgeon’s practice needs to be assessed.
